# The metagenomic and whole-genome metagenomic detection of multidrug-resistant bacteria from subclinical mastitis-affected cow’s milk in India

**DOI:** 10.3389/fcimb.2025.1549523

**Published:** 2025-04-22

**Authors:** Ayyasamy Manimaran, Perumal Arumugam Desingu, Arumugam Kumaresan, Poonam Singh, Komala Subramanya, Pramod Dodamani, Parmar Ankitkumar Dineshbhai

**Affiliations:** ^1^ Southern Regional Station, Indian Council of Agricultural Research (ICAR)-National Dairy Research Institute (NDRI), Bengaluru, Karnataka, India; ^2^ Institute of Advanced Virology, Bio 360 Life Sciences Park, Trivandrum, Kerala, India

**Keywords:** ESKAPE pathogens, pathogenic bacteria, multidrug-resistant bacteria, mastitis-affected cow’s milk, *E. coli*

## Abstract

Multidrug-resistant (MDR) bacteria in farm animal products threaten human health by causing zoonotic infections. The complete genome sequences of MDR bacteria from subclinical mastitis-affected cow’s milk have not yet been comprehensively documented in India. In this study, using a bacterial metagenomic sequencing approach, we detected the nearly complete genome sequences of *Pseudomonas veronii* and *Staphylococcus xylosu*s from subclinical mastitis-affected cow’s milk. Furthermore, we sequenced the nearly complete genome sequences of *Escherichia coli*, *Klebsiella pneumoniae*, *Staphylococcus hominis*, and *S. xylosus* from subclinical mastitis-affected cow’s milk using a whole-genome metagenomic sequencing method. Our analysis subsequently revealed that the complete genome sequences of the identified bacteria contained MDR genes and genes for multiple virulence factors. These MDR bacteria may pose a public health risk through exposure to milkers, milk handlers, and farm workers or through the handling and consumption of unpasteurized milk.

## Introduction

Recently, multidrug-resistant (MDR) bacteria have been detected in cow’s milk in many parts of the world (Tempini et al., 2018; Yang et al., 2021; Hassani et al., 2022; Ashraf et al., 2023; Badawy et al., 2023; Haq et al., 2024), and this poses a threat to public health. Among the MDR bacteria, the ESKAPE pathogens, which include *Enterococcus faecium*, *Staphylococcus aureus*, *Klebsiella pneumoniae*, *Acinetobacter baumannii*, *Pseudomonas aeruginosa*, and *Enterobacter* spp., are emerging as a public health crisis (De Oliveira et al., 2020; Miller and Arias, 2024). The environmental reservoirs and contaminated food of animal or plant origin are considered a source of ESKAPE pathogens spreading to humans (Patil et al., 2021). Continuous use/misuse/overuse of antibiotics in clinical and non-clinical settings, animals, plants, and environments is considered to be the driving force for the emergence of antibiotic-resistant bacteria (Pillay et al., 2022).

In recent years, the complete genome sequencing of bacteria using next-generation sequencing (NGS) has emerged as a powerful tool for monitoring emerging pathogens, particularly those with virulence and antibiotic resistance genes (Boolchandani et al., 2019; Pillay et al., 2022). This approach enables researchers to identify emerging pathogens in various geographical locations and to trace their sources of dissemination (Boolchandani et al., 2019; Pillay et al., 2022). As a result, it assists in the development of diagnostics and preventive and control measures and the formulation of policies for the judicious use of antibiotics (Boolchandani et al., 2019; Pillay et al., 2022). In line with this, recently, the complete genome sequence of *S. aureus* and non-*aureus* staphylococci and mammaliicocci (NASM) from clinical mastitis bovine milk in India was identified using the NGS approach, and the MDR genes in them were determined (Sivakumar et al., 2023; Ramesh et al., 2024). Moreover, virulence and MDR genes were identified through complete genome sequencing of *S. aureus* collected from the milk of cows infected with subclinical mastitis in South Africa (Khasapane et al., 2024), Rwanda (Ndahetuye et al., 2021), and Brazil (Pizauro et al., 2019). Similarly, the complete genome sequences of *Escherichia coli*, *K. pneumoniae*, and *Bordetella bronchiseptica* with MDR genes have been determined in subclinical mastitis-affected cow’s milk in Bangladesh (Anika et al., 2023), Egypt (Tartor et al., 2021), and New Zealand (Munn et al., 2025), respectively. In this context, the complete genome sequencing of bacterial pathogens with MDR and virulent genes in subclinical mastitis-affected cow’s milk is not fully documented in India. In this context, the present study aimed to utilize the NGS approach to determine the complete genome sequences of bacterial pathogens found in subclinical mastitis-affected cow’s milk in India. Additionally, the study planned to identify MDR genes and virulence genes from the complete genome sequences of these bacteria.

## Materials and methods

### Sample collection and subclinical and clinical mastitis detection using the California mastitis test

The clinical examination of animal, udder, and milk was carried out for the diagnosis of clinical and subclinical mastitis by the veterinary clinician, and milk samples from subclinical mastitis-affected cows were collected from Devanahalli taluk of Bengaluru rural district of Karnataka and Livestock Research Centre, Southern Regional Station of the Indian Council of Agricultural Research (ICAR)-National Dairy Research Institute (NDRI), Bengaluru. Changes in milk (e.g., watery, bloody, curd-like) and udder parenchyma (swelling and redness), with or without systemic symptoms like inappetence and fever, were considered clinical mastitis-affected animals. Animals diagnosed with clinical mastitis by dairy farmers were treated and recorded by veterinarians as per standard practices. In the case of subclinical mastitis, milk and the udder appeared normal, but somatic cell counts (SCCs) increased in milk, which were indirectly detected through the California mastitis test (CMT). The CMT was carried out as per the previous standardized protocols (Dingwell et al., 2003; Rust et al., 2021). Briefly, an equal volume of milk and the CMT reagent was mixed in the CMT paddle, and the mixture was rotated in the horizontal position. The mixture with normal fluidity and no thickening was considered healthy, while the mixture with thickening or gel formation was considered subclinical mastitis-affected.

### The bacterial culture; Gram staining; and indole, methyl red, Voges-Proskauer, and citrate tests

The milk samples positive for the CMT were subjected to bacterial isolation in MacConkey agar [M7408; M/s HiMedia Laboratories Pvt. Ltd., Thane (West), Maharashtra, India], Edwards Medium Base [M748; M/s HiMedia Laboratories Pvt. Ltd., Thane (West), Maharashtra, India], and mannitol salt agar (MSA) [MU118; M/s HiMedia Laboratories Pvt. Ltd., Thane (West), Maharashtra, India] as per the previous standard protocol (Sawant et al., 2002; Gebremedhin et al., 2022; Geletu et al., 2022). Then, bacterial colonies grown in MacConkey agar, Edwards Medium Base, and MSA were used for Gram staining characterization. Bacterial Gram staining was performed using the Gram stains kit [K001; M/s HiMedia Laboratories Pvt. Ltd., Thane (West), Maharashtra, India] as per the manufacturer’s protocols. The indole, methyl red, Voges-Proskauer, citrate + and H_2_S (IMViC) [KB001; M/s HiMedia Laboratories Pvt. Ltd., Thane (West), Maharashtra, India] biochemical tests were performed to differentially identify the *Enterobacteriaceae* bacteria with standard protocols (https://microbenotes.com/imvic-tests/).

### The metagenomic and whole-genome metagenomic sequencing of bacteria and analysis

For metagenomic sequencing, DNA was extracted from the subclinical mastitis-affected cow’s milk. Then, preparation of the bacterial metagenomic DNA library was carried out using the QIASeq FX DNA Library (Cat#180475; QIAGEN, Hilden, Germany) protocol as follows: enzymatic fragmentation, end-repair, 3′ adenylation, adapter ligation, six cycles of indexing-PCR, and purification using CamSelect magnetic beads. Sequencing was carried out using NovaSeq 6000. The whole-genome metagenomic sequencing of bacteria was also performed similar to the bacterial metagenomic protocol, but the DNA used for sequencing was extracted from the bacterial culture. The quality control of the sequences was carried out using FastQC (version 0.11.5). Then, Trimmomatic (Bolger et al., 2014) was used for quality control to filter, identify, and remove potential adapters and low-quality read sequences. The bacteria-specific reads were filtered using the protein-based alignment method DIAMOND (Buchfink et al., 2015) and subsequently *de-novo* assembled in the metaSPAdes (Bankevich et al., 2012). The bacteria-specific sequences in the *de-novo* assembled sequences were recognized by Blastx and Blastn in the NCBI RefSeq database for bacteria, and the bacteria-specific contig was aligned using an advanced genome aligner (AGA) (Deforche, 2017), via the Needleman–Wunsch (Smith and Waterman, 1981), Gotoh (1982), and Smith–Waterman algorithms (Smith and Waterman, 1981). Finally, the variant caller GATK/BCFtools (https://github.com/broadinstitute/gatk/releases; https://samtools.github.io/bcftools/howtos/variant-calling.html) was used for optimal alignment and consensus.

### Bacterial genome annotation and detection of antibiotic resistance and virulence factor genes

The sequences of the nearly complete genome of bacteria detected by the NGS approach were genome annotated with Bakta v1.8.2 (DB: v5.0 - Light) (Schwengers et al., 2021) and Prokka 1.14.6 (Seemann, 2014) and visualized in Proksee (Grant et al., 2023). Then, the antibiotic resistance genes in these bacteria were determined using the Comprehensive Antibiotic Resistance Database (CARD) and the Resistance Gene Identifier (RGI) 6.0.3 (Alcock et al., 2023), and the results were validated using the NCBI public database. Furthermore, the virulence factor genes in these bacteria were determined using the Virulence Factor Database (VFDB) (Liu et al., 2022).

## Results

**Figure 1:**
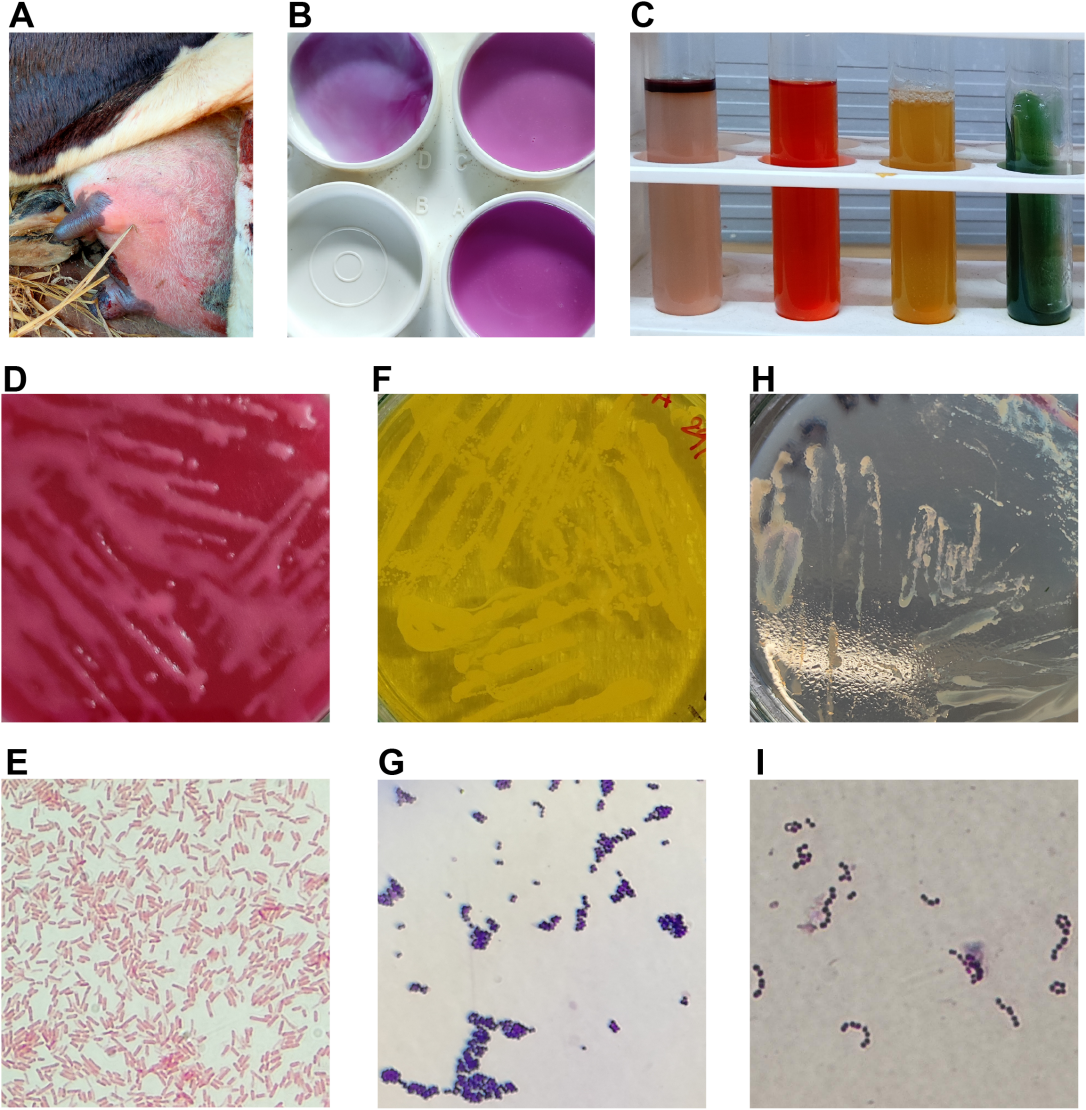
**(A)** Cow with clinical mastitis-affected udder. **(B)** Formation of gel in the paddle containing an equal amount of milk and CMT solution, indicating the SCM in the left front quarter of the cow. **(C)** IMViC tests show positive for the indole and methyl red tests and negative for the remaining tests. It is indicative of *Escherichia coli*-positive. **(D)** Pink colonies of *E coli* growth on MacConkey agar. **(E)** Pink color rod-shaped *E coli* in Gram staining. **(F)** Yellow color *Staphylococcus* spp. colony growth in MSA. **(G)** Bunch of grape-like purple color *Staphylococcus* spp. cocci in Gram staining. **(H)** Colorless *Streptococcus* spp. colony growth in Edward’s medium. **(I)** Purple color chains of *Streptococcus* spp. cocci in Gram staining.

In this study, we documented both clinical and subclinical cases of mastitis that occurred in Bengaluru, Karnataka, India, from 2017 to 2020 (**Table 1**). Accordingly, it was revealed that clinical mastitis cases occurred at almost the same rate in the Bengaluru region in all the years of the study periods and different seasons of the year (**Table 1**). It was also revealed that subclinical mastitis cases were more common than clinical mastitis cases in this region (**Table 1**). In cases of clinical mastitis, farmers observed distinct abnormalities in the udder (**Figure 1A**) as well as noticeable changes in the milk. These conditions were typically addressed through appropriate treatments administered by veterinarians. Conversely, subclinical mastitis may not show visible signs in the udder or milk, necessitating diagnosis by a veterinary clinician. As a result, most mastitis cases were subclinical and were often undetected in the rural areas of India. Handling milk from cows with subclinical mastitis can elevate the risk of spreading infectious agents to farm workers, and the sale of unpasteurized milk to households poses a public health threat. Despite this, traditional hand milking persists in rural India, where it is common to give newborns or children locally available, unpackaged boiled cow’s milk instead of pasteurized milk (personal observation). Therefore, this study aimed to identify antibiotic-resistant bacteria using a metagenomic approach in cow’s milk affected by subclinical mastitis, which presents a health hazard in the country.

**Table 1:** Clinical and subclinical mastitis cases recorded from 2017 to 2020 in Bengaluru, Karnataka, India.

Year	2017			2018			2019			2020		
No. of clinical mastitis	6,017			5,016			7,622			8,120		
Season	Rainy	Winter	Summer	Rainy	Winter	Summer	Rainy	Winter	Summer	Rainy	Winter	Summer
	2,184	1,846	1,987	1,522	1,732	1,762	2,822	2,484	2,316	3,057	2,425	2,638
No. of CMT tested^a^	13,713			16,328			14,529			15,391		
No. of CMT positive	11,329			14,201			11,190			12,478		

a The suspected case of subclinical mastitis was tested for confirmation using the CMT.

The present study results indicated that most clinically suspected cases of subclinical mastitis in apparently healthy animals, as identified by veterinarians, tested positive for the CMT (**Table 1**; **Figure 1B**). Bacteria were subsequently isolated from the cow’s milk with subclinical mastitis on MacConkey agar, with Gram staining and IMViC tests performed, suggesting the presence of *E. coli* in the milk (**Figures 1C–E**). Likewise, bacteria isolated on Edwards Medium Base and MSA, followed by Gram staining, suggested the likely presence of *Streptococcus* spp. and *Staphylococcus* spp., respectively (**Figures 1F–I**). In light of these findings, we employed a bacterial metagenomic method to explore further additional bacteria present in the milk from cows with subclinical mastitis. Through this approach, we discovered nearly complete genomes of *Pseudomonas veronii* and *Staphylococcus xylosus* (**Supplementary Data Sheet 1**). Next, we proceeded with whole-genome sequencing of bacteria isolated from MacConkey agar, Edwards Medium Base, and MSA. The bacterial whole metagenomic sequencing yielded nearly complete genomes for *E. coli*, *K. pneumoniae*, *Staphylococcus hominis*, and *S. xylosus* (**Supplementary Data Sheet 1**).

**Figure 2:**
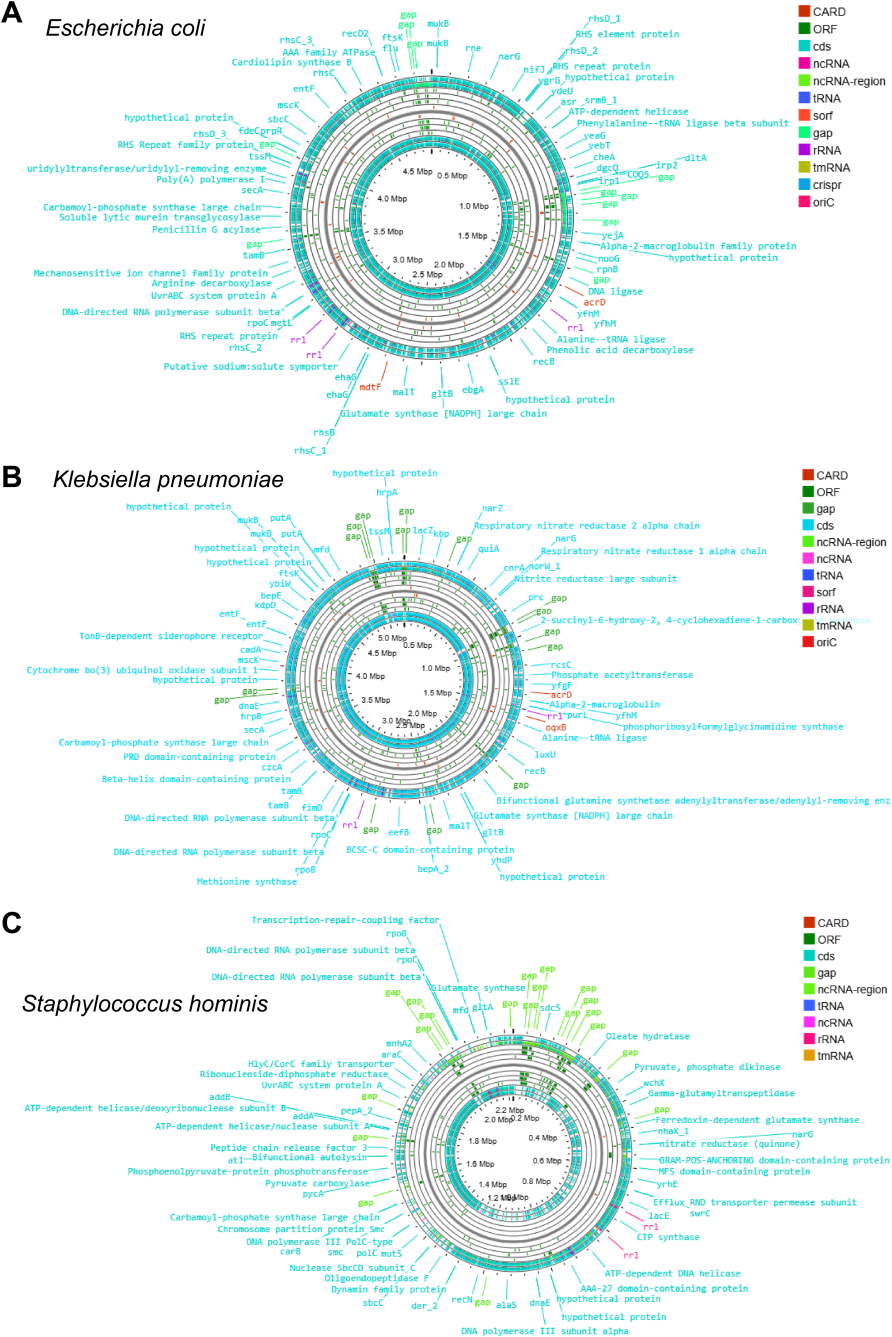
The circular genome map depicting the ORFs, CARDs, rRNAs, ncRNAs, and tRNAs of **(A)** *Escherichia coli*, **(B)** *Klebsiella pneumoniae*, and **(C)** *Staphylococcus hominis*.

**Figure 3:**
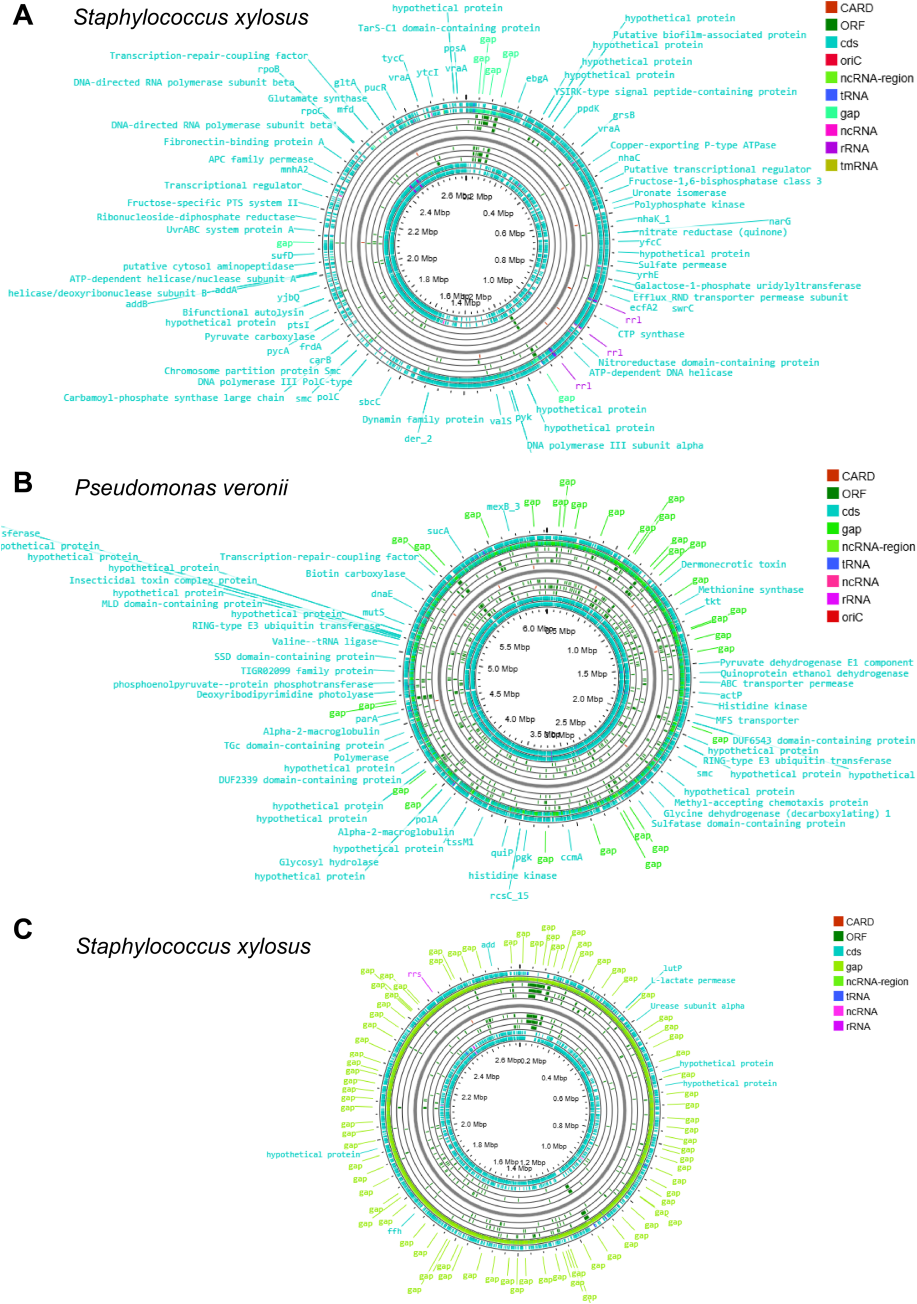
The circular genome map depicting the ORFs, CARDs, rRNAs, ncRNAs, and tRNAs of **(A)** *Staphylococcus xylosus*, **(B)** *Pseudomonas veronii*, and **(C)** *Staphylococcus xylosus* (detected using the metagenomic approach).

The genome annotation of these sequences revealed numerous open reading frames (ORFs) in *E. coli* (**Figure 2A**), *K. pneumoniae* (**Figure 2B**), *S. hominis* (**Figure 2C**), *S. xylosus* (**Figure 3A**), *P. veronii* (**Figure 3B**), and *S. xylosus* (detected by the metagenomic approach) (**Figure 3C**). The nearly complete genomes were also used to predict antibiotic-resistant genes. Specifically, *E. coli* exhibited 320 antibiotic resistance genes, categorized as perfect (12), strict (41), and loose (267) matches in the CARD analysis (**Table 2**; **Supplementary Data Sheet 2**). Notably, *E. coli* displayed multiple MDR genes with perfect and strict matches, including those related to carbapenem, penam, cephalosporin, macrolide, cephamycin, tetracycline, aminoglycoside, aminocoumarin, fluoroquinolone, rifamycin, elfamycin, nitroimidazole, phosphonic acid, monobactam, glycylcycline, phenicol, lincosamide, diaminopyrimidine, nucleoside antibiotic, peptide antibiotic, disinfecting agents, and antiseptics (**Table 2**; **Supplementary Data Sheet 2**). Additionally, the detected *E. coli* showed a single nucleotide polymorphism (SNP) in AcrAB-TolC with MarR mutations that confer resistance to ciprofloxacin and tetracycline associated with Y137H and G103S (**Table 2**; **Supplementary Data Sheet 2**). Other mutations were identified, including those in *E. coli* EF-Tu linked to pulvomycin resistance (R234F), mutations in *Haemophilus influenzae* PBP3 affecting beta-lactam antibiotics (D350N, S357N), and *E. coli* GlpT mutations conferring resistance to fosfomycin (E448K) (**Table 2**; **Supplementary Data Sheet 2**). The plasmid sequence identified in *E. coli* showed a TEM beta-lactamase and tet(A) tetracycline antibiotic resistance genes (**Table 2**; **Supplementary Data Sheet 2**).

**Table 2:** A comprehensive depiction of the antibiotic resistance ontology (ARO), including SNPs, AMR gene families, drug classes, and their corresponding resistance mechanisms in the identified bacteria.

*Escherichia coli*					
RGI Criteria	ARO term	SNP	AMR gene family	Drug Class	Resistance mechanism
Strict	*Escherichia coli* AcrAB-TolC with MarR mutations conferring resistance to ciprofloxacin and tetracycline	Y137H, G103S	Resistance-nodulation-cell division (RND) antibiotic efflux pump	Fluoroquinolone antibiotic, cephalosporin, glycylcycline, penam, tetracycline antibiotic, rifamycin antibiotic, phenicol antibiotic, disinfecting agents, and antiseptics	Antibiotic target alteration, antibiotic efflux
Strict	*Klebsiella pneumoniae* KpnF		Small multidrug resistance (SMR) antibiotic efflux pump	Macrolide antibiotic, aminoglycoside antibiotic, cephalosporin, tetracycline antibiotic, peptide antibiotic, rifamycin antibiotic, disinfecting agents, and antiseptics	Antibiotic efflux
Strict	*Klebsiella pneumoniae* KpnE		Small multidrug resistance (SMR) antibiotic efflux pump	Macrolide antibiotic, aminoglycoside antibiotic, cephalosporin, tetracycline antibiotic, peptide antibiotic, rifamycin antibiotic, disinfecting agents, and antiseptics	Antibiotic efflux
Strict	mdtA		Resistance-nodulation-cell division (RND) antibiotic efflux pump	Aminocoumarin antibiotic	Antibiotic efflux
Strict	mdtB		Resistance-nodulation-cell division (RND) antibiotic efflux pump	Aminocoumarin antibiotic	Antibiotic efflux
Strict	mdtC		Resistance-nodulation-cell division (RND) antibiotic efflux pump	Aminocoumarin antibiotic	Antibiotic efflux
Strict	baeR		Resistance-nodulation-cell division (RND) antibiotic efflux pump	Aminoglycoside antibiotic, aminocoumarin antibiotic	Antibiotic efflux
Strict	YojI		ATP-binding cassette (ABC) antibiotic efflux pump	Peptide antibiotic	Antibiotic efflux
Strict	PmrF		pmr phosphoethanolamine transferase	Peptide antibiotic	Antibiotic target alteration
Strict	ArnT		pmr phosphoethanolamine transferase	Peptide antibiotic	Antibiotic target alteration
Strict	emrY		Major facilitator superfamily (MFS) antibiotic efflux pump	Tetracycline antibiotic	Antibiotic efflux
Strict	emrK		Major facilitator superfamily (MFS) antibiotic efflux pump	Tetracycline antibiotic	Antibiotic efflux
Strict	evgS		Major facilitator superfamily (MFS) antibiotic efflux pump, resistance-nodulation-cell division (RND) antibiotic efflux pump	Macrolide antibiotic, fluoroquinolone antibiotic, penam, tetracycline antibiotic	Antibiotic efflux
Strict	acrD		Resistance-nodulation-cell division (RND) antibiotic efflux pump	Aminoglycoside antibiotic	Antibiotic efflux
Strict	emrA		Major facilitator superfamily (MFS) antibiotic efflux pump	Fluoroquinolone antibiotic	Antibiotic efflux
Strict	rsmA		Resistance-nodulation-cell division (RND) antibiotic efflux pump	Fluoroquinolone antibiotic, diaminopyrimidine antibiotic, phenicol antibiotic	Antibiotic efflux
Strict	TolC		ATP-binding cassette (ABC) antibiotic efflux pump, major facilitator superfamily (MFS) antibiotic efflux pump, resistance-nodulation-cell division (RND) antibiotic efflux pump	macrolide antibiotic, fluoroquinolone antibiotic, aminoglycoside antibiotic, carbapenem, cephalosporin, glycylcycline, cephamycin, penam, tetracycline antibiotic, peptide antibiotic, aminocoumarin antibiotic, rifamycin antibiotic, phenicol antibiotic, penem, disinfecting agents, and antiseptics	Antibiotic efflux
Strict	bacA		Undecaprenyl pyrophosphate-related proteins	Peptide antibiotic	Antibiotic target alteration
Strict	AcrS		Resistance-nodulation-cell division (RND) antibiotic efflux pump	Fluoroquinolone antibiotic, cephalosporin, glycylcycline, cephamycin, penam, tetracycline antibiotic, rifamycin antibiotic, phenicol antibiotic, disinfecting agents, and antiseptics	Antibiotic efflux
Strict	AcrF		Resistance-nodulation-cell division (RND) antibiotic efflux pump	Fluoroquinolone antibiotic, cephalosporin, cephamycin, penam	Antibiotic efflux
Strict	*Escherichia coli* EF-Tu mutants conferring resistance to pulvomycin	R234F	Elfamycin-resistant EF-Tu	Elfamycin antibiotic	Antibiotic target alteration
Strict	CRP		Resistance-nodulation-cell division (RND) antibiotic efflux pump	Macrolide antibiotic, fluoroquinolone antibiotic, penam	Antibiotic efflux
Strict	mdtE		Resistance-nodulation-cell division (RND) antibiotic efflux pump	Macrolide antibiotic, fluoroquinolone antibiotic, penam	Antibiotic efflux
Strict	mdtF		Resistance-nodulation-cell division (RND) antibiotic efflux pump	Macrolide antibiotic, fluoroquinolone antibiotic, penam	Antibiotic efflux
Strict	gadX		Resistance-nodulation-cell division (RND) antibiotic efflux pump	Macrolide antibiotic, fluoroquinolone antibiotic, penam	Antibiotic efflux
Strict	*Escherichia coli* EF-Tu mutants conferring resistance to pulvomycin	R234F	Elfamycin-resistant EF-Tu	Elfamycin antibiotic	Antibiotic target alteration
Strict	*Escherichia coli* soxS with mutation conferring antibiotic resistance		ATP-binding cassette (ABC) antibiotic efflux pump, major facilitator superfamily (MFS) antibiotic efflux pump, resistance-nodulation-cell division (RND) antibiotic efflux pump, general bacterial porin with reduced permeability to beta-lactams	Fluoroquinolone antibiotic, monobactam, carbapenem, cephalosporin, glycylcycline, cephamycin, penam, tetracycline antibiotic, rifamycin antibiotic, phenicol antibiotic, penem, disinfecting agents, and antiseptics	Antibiotic target alteration, antibiotic efflux, reduced permeability to antibiotic
Strict	*Escherichia coli* soxR with mutation conferring antibiotic resistance		ATP-binding cassette (ABC) antibiotic efflux pump, major facilitator superfamily (MFS) antibiotic efflux pump, resistance-nodulation-cell division (RND) antibiotic efflux pump	Fluoroquinolone antibiotic, cephalosporin, glycylcycline, penam, tetracycline antibiotic, rifamycin antibiotic, phenicol antibiotic, disinfecting agents, and antiseptics	Antibiotic target alteration, antibiotic efflux
Strict	mdtP		Major facilitator superfamily (MFS) antibiotic efflux pump	Nucleoside antibiotic, disinfecting agents, and antiseptics	Antibiotic efflux
Strict	mdtO		Major facilitator superfamily (MFS) antibiotic efflux pump	Nucleoside antibiotic, disinfecting agents, and antiseptics	Antibiotic efflux
Strict	mdtN		Major facilitator superfamily (MFS) antibiotic efflux pump	Nucleoside antibiotic, disinfecting agents, and antiseptics	Antibiotic efflux
Strict	eptA		pmr phosphoethanolamine transferase	Peptide antibiotic	Antibiotic target alteration
Strict	EC-13		EC beta-lactamase	Cephalosporin	Antibiotic inactivation
Strict	mdtM		Major facilitator superfamily (MFS) antibiotic efflux pump	Fluoroquinolone antibiotic, lincosamide antibiotic, nucleoside antibiotic, phenicol antibiotic, disinfecting agents, and antiseptics	Antibiotic efflux
Strict	leuO		Major facilitator superfamily (MFS) antibiotic efflux pump	Nucleoside antibiotic, disinfecting agents, and antiseptics	Antibiotic efflux
Strict	*Haemophilus influenzae* PBP3 conferring resistance to beta-lactam antibiotics	D350N, S357N	Penicillin-binding protein mutations conferring resistance to beta-lactam antibiotics	Cephalosporin, cephamycin, penam	Antibiotic target alteration
Strict	vanG		glycopeptide resistance gene cluster, Van ligase	Glycopeptide antibiotic	Antibiotic target alteration
Strict	*Escherichia coli* AcrAB-TolC with AcrR mutation conferring resistance to ciprofloxacin, tetracycline, and ceftazidime		Resistance-nodulation-cell division (RND) antibiotic efflux pump	Fluoroquinolone antibiotic, cephalosporin, glycylcycline, penam, tetracycline antibiotic, rifamycin antibiotic, phenicol antibiotic, disinfecting agents, and antiseptics	Antibiotic target alteration, antibiotic efflux
Strict	kdpE		kdpDE	Aminoglycoside antibiotic	Antibiotic efflux
Strict	*Escherichia coli* mdfA		Major facilitator superfamily (MFS) antibiotic efflux pump	Tetracycline antibiotic, phenicol antibiotic, disinfecting agents, and antiseptics	Antibiotic efflux
Strict	*Escherichia coli* GlpT with mutation conferring resistance to fosfomycin	E448K	Antibiotic-resistant GlpT	Phosphonic acid antibiotic	Antibiotic target alteration
Perfect	msbA		ATP-binding cassette (ABC) antibiotic efflux pump	Nitroimidazole antibiotic	Antibiotic efflux
Perfect	mdtG		Major facilitator superfamily (MFS) antibiotic efflux pump	Phosphonic acid antibiotic	Antibiotic efflux
Perfect	mdtH		Major facilitator superfamily (MFS) antibiotic efflux pump	Fluoroquinolone antibiotic	Antibiotic efflux
Perfect	H-NS		Major facilitator superfamily (MFS) antibiotic efflux pump, resistance-nodulation-cell division (RND) antibiotic efflux pump	Macrolide antibiotic, fluoroquinolone antibiotic, cephalosporin, cephamycin, penam, tetracycline antibiotic	Antibiotic efflux
Perfect	marA		Resistance-nodulation-cell division (RND) antibiotic efflux pump, general bacterial porin with reduced permeability to beta-lactams	Fluoroquinolone antibiotic, monobactam, carbapenem, cephalosporin, glycylcycline, cephamycin, penam, tetracycline antibiotic, rifamycin antibiotic, phenicol antibiotic, penem, disinfecting agents, and antiseptics	Antibiotic efflux, reduced permeability to antibiotic
Perfect	evgA		Major facilitator superfamily (MFS) antibiotic efflux pump, resistance-nodulation-cell division (RND) antibiotic efflux pump	Macrolide antibiotic, fluoroquinolone antibiotic, penam, tetracycline antibiotic	Antibiotic efflux
Perfect	emrR		Major facilitator superfamily (MFS) antibiotic efflux pump	Fluoroquinolone antibiotic	Antibiotic efflux
Perfect	emrB		Major facilitator superfamily (MFS) antibiotic efflux pump	Fluoroquinolone antibiotic	Antibiotic efflux
Perfect	AcrE		Resistance-nodulation-cell division (RND) antibiotic efflux pump	Fluoroquinolone antibiotic, cephalosporin, cephamycin, penam	Antibiotic efflux
Perfect	cpxA		Resistance-nodulation-cell division (RND) antibiotic efflux pump	Aminoglycoside antibiotic, aminocoumarin antibiotic	Antibiotic efflux
Perfect	acrB		Resistance-nodulation-cell division (RND) antibiotic efflux pump	Fluoroquinolone antibiotic, cephalosporin, glycylcycline, penam, tetracycline antibiotic, rifamycin antibiotic, phenicol antibiotic, disinfecting agents, and antiseptics	Antibiotic efflux
Perfect	*Escherichia coli* acrA		Resistance-nodulation-cell division (RND) antibiotic efflux pump	Fluoroquinolone antibiotic, cephalosporin, glycylcycline, penam, tetracycline antibiotic, rifamycin antibiotic, phenicol antibiotic, disinfecting agents, and antiseptics	Antibiotic efflux
*Klebsiella pneumoniae*					
Strict	*Klebsiella pneumoniae* KpnE		Small multidrug resistance (SMR) antibiotic efflux pump	Macrolide antibiotic, aminoglycoside antibiotic, cephalosporin, tetracycline antibiotic, peptide antibiotic, rifamycin antibiotic, disinfecting agents, and antiseptics	Antibiotic efflux
Strict	SHV-28		SHV beta-lactamase	Cephalosporin, penam	Antibiotic inactivation
Strict	marA		Resistance-nodulation-cell division (RND) antibiotic efflux pump, general bacterial porin with reduced permeability to beta-lactams	Fluoroquinolone antibiotic, monobactam, carbapenem, cephalosporin, glycylcycline, cephamycin, penam, tetracycline antibiotic, rifamycin antibiotic, phenicol antibiotic, penem, disinfecting agents, and antiseptics	Antibiotic efflux, reduced permeability to antibiotic
Strict	*Escherichia coli* AcrAB-TolC with MarR mutations conferring resistance to ciprofloxacin and tetracycline		Resistance-nodulation-cell division (RND) antibiotic efflux pump, general bacterial porin with reduced permeability to beta-lactams	Fluoroquinolone antibiotic, monobactam, carbapenem, cephalosporin, glycylcycline, cephamycin, penam, tetracycline antibiotic, rifamycin antibiotic, phenicol antibiotic, penem, disinfecting agents, and antiseptics	Antibiotic efflux, reduced permeability to antibiotic
Strict	H-NS		Major facilitator superfamily (MFS) antibiotic efflux pump, resistance-nodulation-cell division (RND) antibiotic efflux pump	Macrolide antibiotic, fluoroquinolone antibiotic, cephalosporin, cephamycin, penam, tetracycline antibiotic	Antibiotic efflux
Strict	baeR		Resistance-nodulation-cell division (RND) antibiotic efflux pump	Aminoglycoside antibiotic, aminocoumarin antibiotic	Antibiotic efflux
Strict	MdtQ		Outer membrane porin (Opr)	Monobactam, carbapenem, cephalosporin, cephamycin, penam, penem	Reduced permeability to antibiotic
Strict	adeF		Resistance-nodulation-cell division (RND) antibiotic efflux pump	Fluoroquinolone antibiotic, tetracycline antibiotic	Antibiotic efflux
Strict	adeF		Resistance-nodulation-cell division (RND) antibiotic efflux pump	Fluoroquinolone antibiotic, tetracycline antibiotic	Antibiotic efflux
Strict	emrR		Major facilitator superfamily (MFS) antibiotic efflux pump	Fluoroquinolone antibiotic	Antibiotic efflux
Strict	*Klebsiella pneumoniae* KpnG		Major facilitator superfamily (MFS) antibiotic efflux pump	Macrolide antibiotic, fluoroquinolone antibiotic, aminoglycoside antibiotic, carbapenem, cephalosporin, penam, peptide antibiotic, penem	Antibiotic efflux
Strict	*Klebsiella pneumoniae* KpnH		Major facilitator superfamily (MFS) antibiotic efflux pump	Macrolide antibiotic, fluoroquinolone antibiotic, aminoglycoside antibiotic, carbapenem, cephalosporin, penam, peptide antibiotic, penem	Antibiotic efflux
Strict	rsmA		Resistance-nodulation-cell division (RND) antibiotic efflux pump	Fluoroquinolone antibiotic, diaminopyrimidine antibiotic, phenicol antibiotic	Antibiotic efflux
Strict	*Escherichia coli* EF-Tu mutants conferring resistance to pulvomycin	R234F	Elfamycin-resistant EF-Tu	Elfamycin antibiotic	Antibiotic target alteration
Strict	CRP		Resistance-nodulation-cell division (RND) antibiotic efflux pump	Macrolide antibiotic, fluoroquinolone antibiotic, penam	Antibiotic efflux
Strict	ArnT		pmr phosphoethanolamine transferase	Peptide antibiotic	Antibiotic target alteration
Strict	eptB		pmr phosphoethanolamine transferase	Peptide antibiotic	Antibiotic target alteration
Strict	*Escherichia coli* EF-Tu mutants conferring resistance to pulvomycin	R234F	Elfamycin-resistant EF-Tu	Elfamycin antibiotic	Antibiotic target alteration
Strict	FosA6		Fosfomycin thiol transferase	Phosphonic acid antibiotic	Antibiotic inactivation
Strict	leuO		Major facilitator superfamily (MFS) antibiotic efflux pump	Nucleoside antibiotic, disinfecting agents, and antiseptics	Antibiotic efflux
Strict	*Haemophilus influenzae* PBP3 conferring resistance to beta-lactam antibiotics	D350N, S357N	Penicillin-binding protein mutations conferring resistance to beta-lactam antibiotics	Cephalosporin, cephamycin, penam	Antibiotic target alteration
Strict	vanG		Glycopeptide resistance gene cluster, Van ligase	Glycopeptide antibiotic	Antibiotic target alteration
Strict	*Shigella flexneri* acrA		Resistance-nodulation-cell division (RND) antibiotic efflux pump	Fluoroquinolone antibiotic, cephalosporin, glycylcycline, penam, tetracycline antibiotic, rifamycin antibiotic, phenicol antibiotic, disinfecting agents, and antiseptics	Antibiotic efflux
Strict	msbA		ATP-binding cassette (ABC) antibiotic efflux pump	Nitroimidazole antibiotic	Antibiotic efflux
Strict	OmpA		General bacterial porin with reduced permeability to peptide antibiotics	Peptide antibiotic	Reduced permeability to antibiotic
Strict	*Klebsiella pneumoniae* OmpK37		General bacterial porin with reduced permeability to beta-lactams	Monobactam, carbapenem, cephalosporin, cephamycin, penam, penem	Reduced permeability to antibiotic
Strict	*Escherichia coli* UhpT with mutation conferring resistance to fosfomycin	E350Q	Antibiotic-resistant UhpT	Phosphonic acid antibiotic	Antibiotic target alteration
Perfect	*Klebsiella pneumoniae* KpnF		Small multidrug resistance (SMR) antibiotic efflux pump	Macrolide antibiotic, aminoglycoside antibiotic, cephalosporin, tetracycline antibiotic, peptide antibiotic, rifamycin antibiotic, disinfecting agents, and antiseptics	Antibiotic efflux
Perfect	oqxA		Resistance-nodulation-cell division (RND) antibiotic efflux pump	Fluoroquinolone antibiotic, glycylcycline, tetracycline antibiotic, diaminopyrimidine antibiotic, nitrofuran antibiotic	Antibiotic efflux
Perfect	LptD		ATP-binding cassette (ABC) antibiotic efflux pump	Carbapenem, peptide antibiotic, aminocoumarin antibiotic, rifamycin antibiotic	Antibiotic efflux
*Staphylococcus hominis*					
Strict	vanY gene in the vanG cluster		vanY, glycopeptide resistance gene cluster	Glycopeptide antibiotic	Antibiotic target alteration
Strict	sdrM		Major facilitator superfamily (MFS) antibiotic efflux pump	Fluoroquinolone antibiotic, disinfecting agents, and antiseptics	Antibiotic efflux
Strict	sepA		Small multidrug resistance (SMR) antibiotic efflux pump	Disinfecting agents and antiseptics	Antibiotic efflux
Strict	vanT gene in vanG cluster		Glycopeptide resistance gene cluster, vanT	Glycopeptide antibiotic	Antibiotic target alteration
*Staphylococcus xylosus*					
Strict	norC		Major facilitator superfamily (MFS) antibiotic efflux pump	Fluoroquinolone antibiotic, disinfecting agents, and antiseptics	Antibiotic efflux
Strict	norC		Major facilitator superfamily (MFS) antibiotic efflux pump	Fluoroquinolone antibiotic, disinfecting agents, and antiseptics	Antibiotic efflux
Strict	sdrM		Major facilitator superfamily (MFS) antibiotic efflux pump	Fluoroquinolone antibiotic, disinfecting agents, and antiseptics	Antibiotic efflux
Strict	sepA		Small multidrug resistance (SMR) antibiotic efflux pump	Disinfecting agents and antiseptics	Antibiotic efflux
Strict	vanT gene in vanG cluster		Glycopeptide resistance gene cluster, vanT	Glycopeptide antibiotic	Antibiotic target alteration
Strict	vanY gene in the vanM cluster		vanY, glycopeptide resistance gene cluster	Glycopeptide antibiotic	Antibiotic target alteration
Strict	salD		sal-type ABC-F protein	Lincosamide antibiotic, streptogramin antibiotic, streptogramin A antibiotic, pleuromutilin antibiotic	Antibiotic target protection
Strict	FosBx1		Fosfomycin thiol transferase	Phosphonic acid antibiotic	Antibiotic inactivation
Strict	vanY gene in the vanM cluster		vanY, glycopeptide resistance gene cluster	Glycopeptide antibiotic	Antibiotic target alteration
Strict	*Staphylococcus aureus* GlpT with mutation conferring resistance to fosfomycin	L27F	Antibiotic-resistant GlpT	Phosphonic acid antibiotic	Antibiotic target alteration
*Pseudomonas veronii*					
Strict	ArnT		pmr phosphoethanolamine transferase	Peptide antibiotic	Antibiotic target alteration
Strict	adeF		Resistance-nodulation-cell division (RND) antibiotic efflux pump	Fluoroquinolone antibiotic, tetracycline antibiotic	Antibiotic efflux
Strict	vanG		Glycopeptide resistance gene cluster, Van ligase	Glycopeptide antibiotic	Antibiotic target alteration
Strict	FosA8		Fosfomycin thiol transferase	Phosphonic acid antibiotic	Antibiotic inactivation
Strict	*Pseudomonas aeruginosa* soxR		ATP-binding cassette (ABC) antibiotic efflux pump, major facilitator superfamily (MFS) antibiotic efflux pump, resistance-nodulation-cell division (RND) antibiotic efflux pump	Fluoroquinolone antibiotic, cephalosporin, glycylcycline, penam, tetracycline antibiotic, rifamycin antibiotic, phenicol antibiotic, disinfecting agents, and antiseptics	Antibiotic target alteration, antibiotic efflux
Strict	*Acinetobacter baumannii* AbaQ		Major facilitator superfamily (MFS) antibiotic efflux pump	Fluoroquinolone antibiotic	Antibiotic efflux
*Staphylococcus xylosus* detected by the metagenomic approach					
Strict	vanY gene in the vanA cluster		vanY, glycopeptide resistance gene cluster	Glycopeptide antibiotic	Antibiotic target alteration

Likewise, the *K. pneumoniae* genome exhibited the following MDR genes with perfect and strict matches: carbapenem, penam, cephalosporin, macrolide, cephamycin, tetracycline, aminoglycoside, aminocoumarin, fluoroquinolone, rifamycin, elfamycin, nitroimidazole, nitrofuran, phosphonic acid, monobactam, glycylcycline, glycopeptide, phenicol, diaminopyrimidine, nucleoside antibiotic, peptide antibiotic, disinfecting agents, and antiseptics (**Table 2**; **Supplementary Data Sheet 2**). Similarly, *K. pneumoniae* also revealed an SNP in EF-Tu mutants that impart resistance to pulvomycin (R234F) and in *H. influenzae* PBP3 correlating with beta-lactam resistance (D350N, S357N). Additionally, a mutation in *E. coli* UhpT was noted, granting resistance to fosfomycin (E350Q) (**Table 2**; **Supplementary Data Sheet 2**).

The *S. hominis* genome indicated resistance genes for glycopeptide, fluoroquinolone, disinfecting agents, and antiseptics (**Table 2**; **Supplementary Data Sheet 2**). The *S. xylosus* genome presented resistance genes for glycopeptide, fluoroquinolone, phosphonic acid, lincosamide, streptogramin, pleuromutilin, disinfecting agents, and antiseptics (**Table 2**; **Supplementary Data Sheet 2**). It also harbored an SNP in the *S. aureus* GlpT conferring resistance to fosfomycin (L27F). The *P. veronii* genome exhibited resistance genes for cephalosporin, penam, tetracycline, rifamycin, fluoroquinolone, glycopeptide, phosphonic acid, glycylcycline, phenicol, disinfecting agents, and antiseptics (**Table 2**; **Supplementary Data Sheet 2**). The *S. xylosus* genome identified through metagenomic analysis contained the glycopeptide antibiotic resistance gene (**Table 2**; **Supplementary Data Sheet 2**).

**Figure 4:**
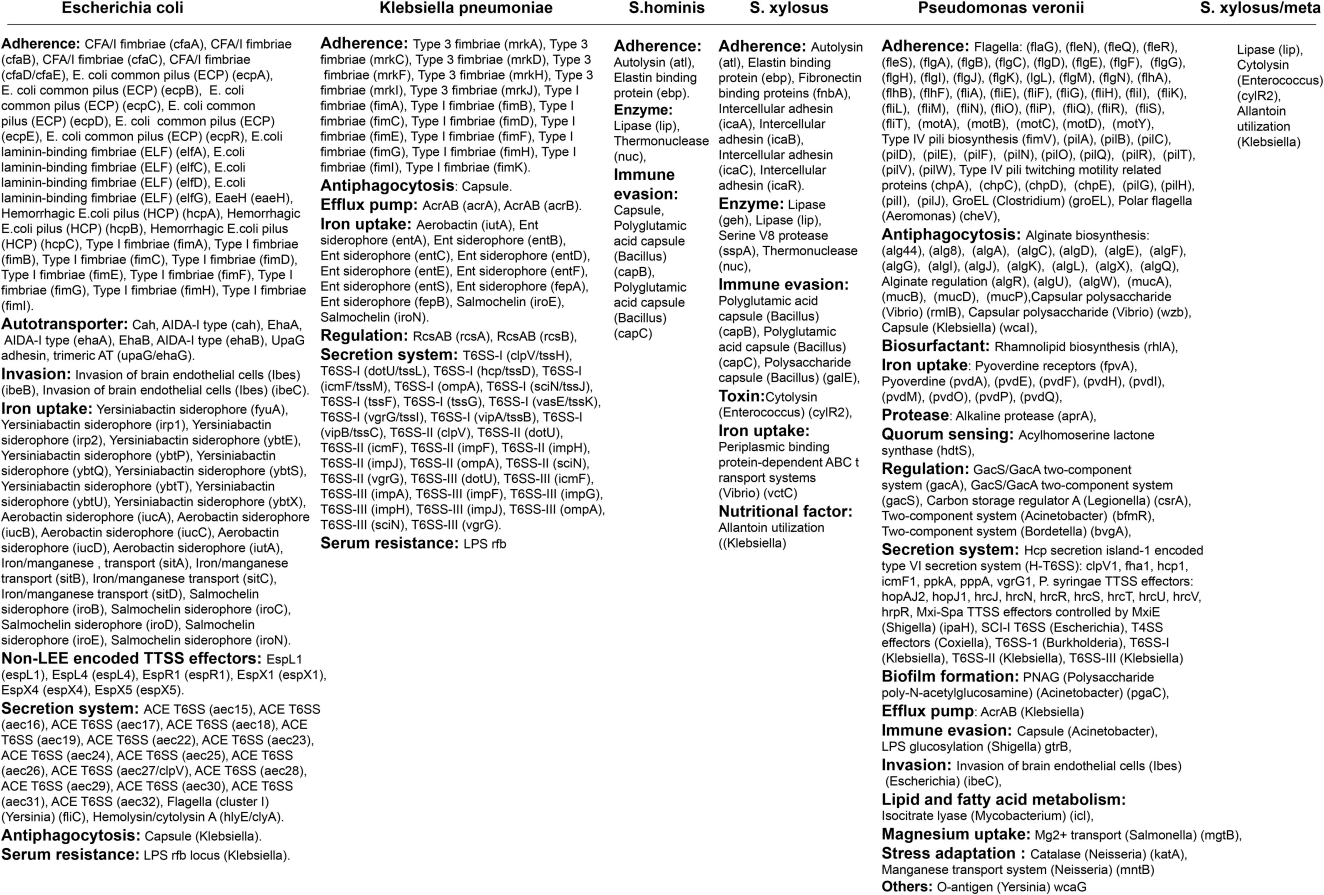
The table represents the virulence genes detected in *Escherichia coli*, *Klebsiella pneumoniae*, *Staphylococcus hominis*, *Staphylococcus xylosus*, and *Pseudomonas veronii* (details are provided in **Supplementary Data Sheet 3**).

Furthermore, the nearly complete genome sequences were utilized to predict the presence of virulence factor genes. The *E. coli* found in the subclinical mastitis-affected cow possessed nearly all virulent genes analogous to those of *Enteroaggregative Escherichia coli* (EAEC) (**Figure 4**; **Supplementary Data Sheet 3**). *Klebsiella pneumoniae* identified in the study exhibited virulent genes similar to *K. pneumoniae* 342/*K. pneumoniae* JM45/*K. pneumoniae* KCTC 2242 (**Figure 4**; **Supplementary Data Sheet 3**). Both *S. hominis* and *S. xylosus* presented virulent genes equivalent to those of *Staphylococcus haemolyticus* JCSC1435 and *Staphylococcus epidermidis* RP62A, respectively (**Figure 4**; **Supplementary Data Sheet 3**). Although *P. veronii* exhibited virulence genes found in other *Pseudomonas* species, the LPS O-antigen (*P. aeruginosa*) was not detected (**Figure 4**; **Supplementary Data Sheet 3**).

## Discussion

In the present study, we detected nearly complete genomes of various virulent and antibiotic-resistant bacterial pathogens, including *E. coli*, *K. pneumoniae*, *P. veronii*, *S. hominis*, and *S. xylosus*, from subclinical mastitis-affected cow’s milk in India. It is worth noting that among the bacteria identified in the present study, *E. coli* and *K. pneumoniae* are members of the ESKAPE pathogens (De Oliveira et al., 2020; Miller and Arias, 2024). Furthermore, *E. coli*, *K. pneumoniae*, and *S. hominis* are pathogenic in humans, but *S. xylosus* and *P. veronii* may not cause significant human infections (Kaur et al., 2010; Brand and Rufer, 2021; Chang et al., 2021; Szemraj et al., 2025).

In the case of treatment of the clinical condition caused by the EAEC bacteria, the widely used/recommended antibiotics are azithromycin, ciprofloxacin, and rifaximin (Hebbelstrup Jensen et al., 2018); however, the detected EAEC bacteria have the resistance gene for all these antibiotics. The EAEC bacteria are the emerging pathogens that cause acute and persistent diarrhea in children and adults in developing countries and cause acute diarrhea in travelers who travel to these regions (Nataro and Kaper, 1998; Durrer et al., 2000; Estrada-Garcia and Navarro-Garcia, 2012; Ellis et al., 2020). Recent studies indicate that only a few virulence factors are conserved in *E. coli* associated with bovine mastitis, and these conserved virulence factors are also found in commensal *E. coli* (Kempf et al., 2016; Alawneh et al., 2020). In Bangladesh, a neighboring country of India, the complete genome of *E. coli* found in clinical and subclinical mastitis bovine milk contains genes for multiple virulence factors (Anika et al., 2023; Rahman et al., 2025), and *E. coli* detected in the present study was also found to have various virulent factor genes. Furthermore, plasmids in *E. coli*, in particular, contain MDR genes (Alawneh et al., 2020). It is noteworthy that the *E. coli* plasmid identified in this present study also contains resistance genes for beta-lactam and tetracycline antibiotics. Beta-lactamase producing *E. coli* poses a global health threat to humans (Bajaj et al., 2016; Mahmud et al., 2020), and the beta-lactamase gene in the *E. coli* plasmid identified in this present study has the potential to spread vertically and horizontally in *E. coli*, posing a health threat. Furthermore, the genome of *E. coli* identified in this study contains 53 resistance genes that confer resistance to various antibiotics. This could potentially lead to health issues in humans if these bacteria spread through milk. Therefore, there is a need for large-scale surveillance to monitor such *E. coli* strains in India.

*Klebsiella pneumoniae* presents significant challenges in healthcare settings, especially for neonates, the elderly, and immunocompromised persons (Bengoechea and Sa Pessoa, 2019; Grubwieser et al., 2023; Bai and Guo, 2024). The identification of *K. pneumoniae* in bovine clinical mastitis across various regions of the world underscores its potential for zoonotic transmission (Munoz et al., 2007; Salauddin et al., 2019; Fu et al., 2022; Zheng et al., 2022; Bai and Guo, 2024). Thus, the World Health Organization (WHO) is giving priority to *K. pneumoniae* that contains carbapenemase genes in the Global Antimicrobial Resistance and Surveillance System on Emerging Antimicrobial Resistance Reporting (GLASS-EAR) (https://www.who.int/emergencies/disease-outbreak-news/item/2024-DON527), and *K. pneumoniae* identified in the present study also contains a carbapenem-resistant gene. The treatment of *K. pneumoniae* is challenging due to its thick capsule; however, it can be treated with cephalosporins, quinolones, and carbapenems (Prince et al., 1997). *Klebsiella pneumoniae* detected in the subclinical mastitis-affected cow’s milk in this study has capsules and carries antibiotic resistance genes for all these antibiotics. Additionally, the genome of *K. pneumoniae* identified in this study carries 30 resistance genes that confer resistance to various antibiotics, potentially posing a public health threat in India.

*Staphylococcus hominis* and *S. xylosus* detected in this study have comparatively lesser numbers of antibiotic resistance genes than *E. coli* and *K. pneumoniae*. Recently, complete genome sequencing of the *S. xylosus* strains K19 and K46, derived from clinical mastitis-affected cow’s milk in India, revealed the presence of the salE and fosBx1 resistance genes, and the *S. xylosus* strain SMG24 was found to have the vanY resistance gene (Ramesh et al., 2024). In the present study, it is noteworthy that *S. xylosus* detected in subclinical mastitis-affected cow’s milk contained resistance genes such as norC, sdrM, sepA, and vanT in the vanG cluster; the vanY gene in the vanM cluster; salD; and FosBx1. In addition, antibiotic-resistant *S. hominis* and *S. xylosus* have been identified in Brazil from subclinical mastitis milk and milkers’ hands, respectively (Pizauro et al., 2019). Furthermore, the identification of the vancomycin-resistant *S. hominis* in endophthalmitis in humans also highlights its importance (Won and Kim, 2013). In this line, multiple strains of the complete genome of *S. hominis* detected from clinical mastitis-affected cow’s milk in India have been found to contain several resistance genes such as sepA, mdeA, norC, sdrM, and vanY in the vanB cluster; msrA; PC1 beta-lactamase (blaZ); and tet(K) (Ramesh et al., 2024). It is important to note that *S. xylosus*, detected in the subclinical mastitis-affected cow’s milk in the present study, contained resistance genes such as the vanY gene in the vanG cluster, the vanT gene in the vanG cluster, and sdrM and sepA. Next, there are no reports of *P. veronii* infecting humans (Mullaeva et al., 2022; Zavala-Meneses et al., 2024). The O antigen of lipopolysaccharides (LPS) in *Pseudomona*s bacteria is crucial for their virulence and pathogenesis (Rocchetta et al., 1999; Lam et al., 2011; Huszczynski et al., 2019; Azimi et al., 2021). However, as this study found that *P. veronii* lacks the LPS O antigen, it is unlikely to be virulent in humans/animals.

In conclusion, the present study reports the presence of multiple virulent and antibiotic-resistant genes containing bacteria in the subclinical mastitis cases in India, which poses a public health threat. Early detection of subclinical mastitis and subclinical mastitis milk management training are very important for Indian cow farmers. If such training is not provided, these MDR bacteria are likely to spread to humans from hand milking, milk storage and transportation to boiling, and consumption, posing a public health threat. Large-scale surveillance needs to be conducted to determine how MDR bacteria cause subclinical mastitis and how they accumulate MDR genes in subclinical mastitis, and policies for control, prevention, and treatment measures need to be designed accordingly.

## Data Availability

The datasets presented in this study can be found in online repositories. The names of the repository/repositories and accession number(s) can be found in the article/**Supplementary Material**. BioProject ID PRJNA1188839 and PRJNA1189268, Submitted in NCBI.
