# Supplementary material for: The metagenomic and whole-genome metagenomic detection of multidrug-resistant bacteria from subclinical mastitis-affected cow’s milk in India
Source: Front Cell Infect Microbiol. 2025 Apr 22;15:1549523. doi: 10.3389/fcimb.2025.1549523 (PMC12053146; doi:10.3389/fcimb.2025.1549523)
Supplement: Supplementary file 4 [file Table1.docx]

**The metagenomic and whole-genome metagenomic detection of multidrug-resistant bacteria from subclinical mastitis-affected cow’s milk in India**

Ayyasamy Manimaran^1*^, Perumal Arumugam Desingu^2*^, Arumugam Kumaresan^1^, Poonam Singh^1^, Komala Subramanya^1^, Pramod Dodamani^1^, and Parmar Ankitkumar Dineshbhai^1^

^1^Southern Regional Station, ICAR-National Dairy Research Institute (NDRI), Adugodi, Bengaluru - 560 030, Karnataka, India

^2^Institute of Advanced Virology, Bio 360 Life Sciences Park, Thonnakkal, Trivandrum, Kerala-695 317

**Supplementary materials**

**The details of the metagenomic workflow from QC check, assembly, annotation of genes**

The paired end sequencing reads were generated in the Next-generation sequencing in the Fastq format. Basic Statistics (150 bp of the paired end sequencing, and adapter sequences were confirmed), per sequence quality scores, per base sequence quality, sequence length distribution, overrepresented sequences, per base sequence content, per sequence GC content, per base N content, sequence duplication levels, adapter content, and Kmer content quality control were checked in the FastQC (version 0.11.5)^1^. Then the Trimmomatic^2^ was used to remove potential adopters and low-quality read sequences with the options of ILLUMINACLIP and SLIDINGWINDOW. After removing potential adopters and low-quality read sequences using Trimmomatic^2^ the quality of the reads was once again checked in the FastQC (version 0.11.5)^1^. The quality control passes reads were used for DNA sequence alignment based on the protein reference database using DIAMOND^3^, with the option of bacteria-specific taxon taxid:2, with the options of alignment mode: blastp; composition-based statistics to remove false positive matches, BLOSUM62, Tantan masking algorithm, Double-indexed, and 10 maximum number of target sequences per query. The bacteria-specific reads identified in the DIAMOND^3^ was used for the de novo assembly by metaSPAdes^4^ to generate the contigs. The metaSPAdes^4^ generated contigs were used for the bacteria-specific contig identification by Blastx and Blastn in the NCBI RefSeq database for bacteria, and the bacteria-specific contig was aligned using an advanced genome aligner (AGA)^5^, by the Needleman–Wunsch^6^, Gotoh^7^ and Smith-Waterman^6^. Finally, variant caller GATK/BcfTools^8,9^ is used for optimal alignment algorithms, consensus. In this process, we determined nearly complete genome sequences of *Escherichia coli*, *Klebsiella pneumoniae*, *Staphylococcus hominis*, and *Staphylococcus xylosus*. The sequences of the nearly complete genome of bacteria detected by the metagenomic or whole genome metagenome approach were genome annotated with Bakta v1.8.2 (DB: v5.0 - Light)^10^, and Prokka 1.14.6^11^ and visualized in Proksee^12^. Then the antibiotic resistance genes in these bacteria were determined using the Comprehensive Antibiotic Resistance Database (CARD) and the Resistance Gene Identifier (RGI) RGI 6.0.3^13^, and these results were validated using the NCBI public database. Further, the virulence factor genes in these bacteria were determined using the Virulence Factor Database (VFDB)^14^.

**References**

1 <https://www.bioinformatics.babraham.ac.uk/projects/fastqc/>.

2 Bolger, A. M., Lohse, M. & Usadel, B. Trimmomatic: a flexible trimmer for Illumina sequence data. *Bioinformatics* **30**, 2114-2120, doi:10.1093/bioinformatics/btu170 (2014).

3 Buchfink, B., Xie, C. & Huson, D. H. Fast and sensitive protein alignment using DIAMOND. *Nature methods* **12**, 59-60, doi:10.1038/nmeth.3176 (2015).

4 Bankevich, A. *et al.* SPAdes: a new genome assembly algorithm and its applications to single-cell sequencing. *Journal of computational biology : a journal of computational molecular cell biology* **19**, 455-477, doi:10.1089/cmb.2012.0021 (2012).

5 K, D. An alignment method for nucleic acid sequences against annotated genomes. *bioRxiv*, doi:doi: 10.1101/200394 (2017).

6 Smith, T. F. & Waterman, M. S. Identification of common molecular subsequences. *Journal of molecular biology* **147**, 195-197, doi:10.1016/0022-2836(81)90087-5 (1981).

7 Gotoh, O. An improved algorithm for matching biological sequences. *Journal of molecular biology* **162**, 705-708, doi:10.1016/0022-2836(82)90398-9 (1982).

8 <https://github.com/broadinstitute/gatk/releases>.

9 <https://samtools.github.io/bcftools/howtos/variant-calling.html>.

10 Schwengers, O. *et al.* Bakta: rapid and standardized annotation of bacterial genomes via alignment-free sequence identification. *Microb Genom* **7**, doi:10.1099/mgen.0.000685 (2021).

11 Seemann, T. Prokka: rapid prokaryotic genome annotation. *Bioinformatics* **30**, 2068-2069, doi:10.1093/bioinformatics/btu153 (2014).

12 Grant, J. R. *et al.* Proksee: in-depth characterization and visualization of bacterial genomes. *Nucleic Acids Res* **51**, W484-W492, doi:10.1093/nar/gkad326 (2023).

13 Alcock, B. P. *et al.* CARD 2023: expanded curation, support for machine learning, and resistome prediction at the Comprehensive Antibiotic Resistance Database. *Nucleic Acids Res* **51**, D690-D699, doi:10.1093/nar/gkac920 (2023).

14 Liu, B., Zheng, D., Zhou, S., Chen, L. & Yang, J. VFDB 2022: a general classification scheme for bacterial virulence factors. *Nucleic Acids Res* **50**, D912-D917, doi:10.1093/nar/gkab1107 (2022).
